# Factors affecting clinical manifestation of chromosomal imbalance in carriers of segmental autosomal mosaicism: differential impact of gender

**DOI:** 10.1007/s13353-021-00673-w

**Published:** 2022-01-01

**Authors:** Natalia V. Kovaleva, Philip D. Cotter

**Affiliations:** 1 Academy of Molecular Medicine, Mytninskaya str., 12/44, St. Petersburg, 191144 Russian Federation; 2 ResearchDx Inc., Irvine, CA USA

**Keywords:** Segmental autosomal mosaicism, Unbalanced chromosomal rearrangements, Balanced chromosomal rearrangements, Maternal age, Paternal age, Sex ratio, Gonadal mosaicism, Postnatal diagnosis, Prenatal diagnosis, Preimplantation diagnosis, Miscarriage

## Abstract

Mosaicism for unbalanced chromosomal rearrangements segmental mosaicism (SM) is rare, both in patients referred for cytogenetic testing and in prenatal diagnoses. In contrast, in preimplantation embryos SM is a frequent finding and, therefore, is even more challenging. However, there is no consistency among results of published studies on the clinical outcomes of embryos with SM, primarily due to the small number of reported cases. Moreover, there is the problem of predicting the potential for the optimal development of a mosaic embryo to a healthy individual. Therefore, we suggested comparing factors predisposing to favorable and poor prognoses, identified in postnatal and prenatal cohorts of SM carriers, with those obtained from studies on preimplantation embryos. We analyzed 580 published cases of SM including (i) postnatally diagnosed affected carriers, (ii) clinically asymptomatic carriers, (iii) prenatally diagnosed carriers, and (iv) miscarriages. We observed a concordance with preimplantation diagnoses regarding the clinical significance of the extent of mosaicism as well as a predominance of deletions over other types of rearrangements. However, there is no concordance regarding excessive involvement of chromosomes 1, 5, and 9 in unbalanced rearrangements and a preferential involvement of larger chromosomes compared to short ones. Paternal age was not found to be associated with SM in postnatally disease-defined individuals. We have identified maternal age and preferential involvement of chromosome 18 in rearrangements associated with clinical manifestations. Male predominance was found among normal pregnancy outcomes and among disease-defined carriers of rearrangements resulting in a gain of genomic material. Female predominance was found among abnormal pregnancy outcomes, among disease-defined carriers of loss and gain/loss rearrangements, and among transmitting carriers of gonadal SM, both affected and asymptomatic. According to data obtained from “post-embryo” studies, clinical manifestations of chromosomal imbalance are associated with a high proportion of abnormal cells, female gender, the type of rearrangement and involved chromosome(s), and maternal age. We believe these data are instructive in the challenging medical genetic counseling of parents faced with no option other than transfer of an embryo with segmental mosaicism.

**Supplementary Information:**

The online version contains supplementary material available at 10.1007/s13353-021-00673-w.

## Background

Mosaicism is the presence of more than one genetically distinct cell line in a single organism that originates from a genetically homogenous zygote. Until recently, mosaicism for chromosomal rearrangement (Rea), i.e., segmental SM, was considered extremely rare both in patients referred for cytogenetic testing and in prenatal diagnoses, and therefore its epidemiology had not been studied. Prior to recent publications (Kovaleva and Cotter 2016, 2017a, b), the basic characteristics such as population frequency, cytogenetic profiles, and sex ratio (SR, male-to-female ratio) in various groups of carriers were not identified.

Segmental mosaicism appears to be more frequent than previously thought. For example, a normal cell line was detected in 6–20% of patients with microscopically determined disease-causing deletions (Niebuhr 1978; Cassidy et al. 1984; Munier et al. 1989; Robinson et al. 2008; Kotzot et al. 2005; Shinawi et al. 2010) and in 10% of patients with a ring chromosome (Guilherme et al. 2013). The application of molecular technologies resulted in the detection of more carriers of segmental mosaicism.

Mosaicism is particularly frequent (up to 30% of the cases) in preimplantation embryos (Munné and Wells 2017). Until recently, mosaic embryos had not been considered for transfer. Greco et al. (2020) were the first to demonstrate that mosaic embryos may have the potential for giving birth to healthy offspring. Among mosaic embryos, one-third of the mosaic cases were segmental mosaics (Liu et al. 2017; Nakhuda et al. 2018; Coll et al. 2020). There is no consistency among results of published studies on the clinical outcomes of segmental mosaicism regarding their capacity to implant and develop to a fetus (Liu et al. 2017; Fragouli et al. 2017; Victor et al. 2019). In addition, there is the problem of predicting the potential for the optimal development of a mosaic embryo to a healthy individual, without physical and mental disorders.

Kahraman and colleagues (Kahraman et al. 2020) stated: “The transfer of mosaic embryos marks a new era in ART and future studies and reports of cases are needed to help guide clinicians to make safe decisions regarding mosaic embryos. PGT-A is widely used for a number of indications and with the introduction of Next Generation Sequencing (NGS) and increased identification of mosaicism, clinicians require more informative data to guide them to make safe decisions when considering transfer of mosaic embryos… Patient counseling regarding mosaic embryo transfer is extremely important. Additional reports and data including postpartum karyotype analysis of the newborns are necessary to provide conclusive decisions.”

Munné and Wells (2017) suggested that the types of mosaicism observed during preimplantation development and those that affect the fetus and the newborn might represent different phenomena. They declared that “This is probably one of the most important questions remaining to be answered.” We suggested that it might be reasonable and helpful to compare factors predisposing to favorable and poor prognoses, identified in postnatal and prenatal cohorts of SM carriers, with those obtained from studies on preimplantation embryos.

The aim of this study was to determine the risk factors for clinical manifestation of chromosomal imbalance based on previously obtained and additional data on the SM carriers. The objectives were to compare types of rearrangement, the proportion of abnormal cells, involvement of specific chromosomes in rearrangements, sex ratio, and parental ages in groups of postnatally disease-diagnosed carriers and clinically asymptomatic carriers, as well as in the group of prenatally diagnosed carriers, stratified by the outcome of pregnancy. The data obtained were intended to be compared with the published results of studies carried out on mosaic embryos.

## Methods

The material for the study was published cases of mosaicism on microscopically detectable autosomal non-centromeric Reas with the presence of a normal cell line, diagnosed by conventional cytogenetics with a resolution of up to 850 bands and/or molecular cytogenetics. Overall, more than one thousand publications had been scanned and 434 articles were selected for the analysis. The cases were identified from various sources including PubMed. The following cases were excluded from the analysis: unknown sex of the carrier; Reas in which both breakpoints are localized in the pericentromeric region (since females predominate among the carriers of such Reas [19, 20] and inherited chromosome instability. Cases of SM with reproductive disorders were extracted only from cytogenetic surveys of couples; reports on exclusively male or female contingents were not considered.

A total of 580 SM carriers were evaluated, including 272 disease-defined carriers of somatic SM and 16 affected carriers of generalized (somatic and gonadal SM), 75 clinically asymptomatic carriers of somatic and/or gonadal SM with offspring with a chromosomal abnormality, 9 carriers identified by chance; 46 clinically asymptomatic carriers of somatic SM with reproductive disorders presumably due to gonadal mosaicism; 130 prenatally diagnosed carriers; 32 cases of fetal deaths. According to Barber (2005), individuals were considered phenotypically affected when any type of phenotypic anomaly was reported even if the etiological role of the chromosome abnormality in the same individual is questionable. The studied variables were those readily available prior to and upon genetic testing, i.e., parental ages, indication for the testing, results of the testing, outcome of the tested pregnancy, and gender of SM carriers. All the cases, along with the data on their chromosome constitution, patient’s age at testing/ascertainment, parental ages at the birth/diagnosis of the proband, the proportion of abnormal cell line(s), and indication for testing are tabulated in Supplemental information files S1–S19. References for files S1–S19 are listed in the S20 file. Rearrangements were classified as a loss, gain, and loss/gain of genomic material. Deletion represented the “loss,” duplication, and additional material was categorized as “gain,” derivative chromosomes, isodicentrics, complex rearrangements, and cases with two abnormal cell lines, one of which with deletion, another one with duplication, were classified as “loss/gain.” In some instances, derivatives and other rearrangements were considered apparent or suggestive “gain” or “loss.” Statistical analysis was performed using programs LePAC (https://eris62.eu/ErisLePAC.html) for estimation of 95% confidence intervals (CI) for proportions and/or their ratios, Fisher’s exact test *p*-value calculator, 2 × 2 and 2 × 3 (https://www.cog-genomics.org/software/stats) for estimation of the mid-*p*-values for the Fisher’s exact tests, and StatXact (https://www.cytel.com/software/statxact) for the homogeneity of contingency tables RxC.

## Results and discussion

### Cytogenetic profile of segmental mosaicism

#### Unbalanced rearrangements

The results of the comparison of the distribution of various types of unbalanced Reas in studied groups of SM are shown in Table 1. The most common type of Rea in all studied groups, except fetal deaths, was the “deletion.” The proportion of deletions in asymptomatic carriers appeared to be the highest (_31_ 46 _62_%) among compared groups while the contribution of “unbalanced translocation” was the least common (_3_ 9 _22_%). Overall, the difference between affected carriers and asymptomatic carriers is not statistically significant. Interestingly, the category “other chromosome rearrangements” is prevalent in fetal deaths (_16_ 38 _64_%) but the difference between asymptomatic carriers and fetal deaths is not statistically significant, probably due to the small sample size of the last group.

**Table 1:** Cytogenetic profiles of mosaicism for unbalanced rearrangement in studied groups

Group	Number of carriers	Type of chromosome rearrangement				
		Deletion	Duplication	Ring	Unbalanced translocation	Other rearrangement
Affected carriers^a^	239	79 (_25_ 33 _41_%)	47 (_14_ 20 _27_%)	53 (_16_ 21 _30_%)^b^	30 (_8 _13 _19_%)	30 (_8 _13 _19_%)
Fetal deaths	24	6 (_8 _25 _53_%)	4 (_4 _17 _43_%)	2 (_1 _8 _31_%)	3 (_2_ 12 _39_%)	9 (_16_ 38 _64_%)
Prenatal diagnosis	96	38 (_27_ 40 _53_%)	13 (_6 _13 _24_%)	16 (_9 _17 _28_%)	11 (_5 _11 _22_%)	18 (_10_ 19 _31_%)
Asymptomatic carriers	66	30 (_30_ 46 _62_%)	10 (_7 _15 _29_%)	12 (_8 _19 _32_%)^d^	7 (_4_ 9 _23_%)	7 (_4 _11 _23_%)
Exact *p*-value		0.15				

^a^Excluding 22 cases of rescued rearrangements and 13 cases of del(13q) associated with retinoblastoma; ^b^58% apparently deleted; ^d^breakpoints were not specified in 50% of the cases. Subscripts are limits of the exact 95% confidence intervals for the multinomial probability parameters

Detailed analysis of cytogenetic profiles in asymptomatic carriers (Table 2) shows a difference between transmitting carriers and those with reproductive disorders in the rate of unbalanced translocations, _7_ 16 _27_% (7 out of 48) versus _0.2_ 5 _22_% (0 out of 14), correspondingly, however again, the samples are too small.

**Table 2:** Cytogenetic profiles of unbalanced segmental mosaicism in asymptomatic carriers

Group	Number of carriers	Deletion	Duplication	Ring	Unbalanced translocation	Other rearrangement
Transmitting carriers	48	20	9	7	7	5
Fortitously identified carriers	4	2	0	1	0	1
Carriers with recurrent miscarriage	8	7	1	0	0	0
Carriers with infertility	6	1	0	4	0	1

#### Balanced rearrangements

In disease-defined patients with somatic SM, carriers of balanced Reas were found, though relatively rare, in 6% (14 of 237 patients). This can be due to various reasons, including chance coincidence, microstructural abnormalities in the rearranged chromosome(s) involved (De Gregori et al. 2007), position effect (Zepeda-Mendoza et al. 2017), or undetected cell line with an unbalanced derivative (Raimondi et al. 1983; Dufke et al. 2003). Among 16 affected patients with generalized (somatic and gonadal) mosaicism, individuals with balanced Reas were not found. Among both fetal deaths and in prenatal diagnoses, there were similar proportions of balanced Rea carriers: 25% (8 out of 32) and 26% (34/130). A remarkable difference was found among asymptomatic carriers, an apparent predominance of balanced Reas over unbalanced Reas in patients with reproductive disorders: _55_ 73 _86_% (22 out of 30) with repeated miscarriage and _38_ 62 _82_%), (10 out of 16) with infertility (statistically nonsignificant, mid-*p* = 0.41), while in transmitting carriers of gonadal mosaicism, the proportion of these Reas was twofold lower: _26_ 37 _48_% (27 out of 74) the difference is statistically significant, mid-*p* = 7•10^−4^. At this stage of our research on the Rea types distributions, we conclude that the main difference between the studied groups is the contribution of balanced Rea, but not unbalanced Rea.

The overwhelming majority of balanced Reas were reciprocal translocations, with a low proportion of inversions. Among asymptomatic carriers of gonadal mosaicism and individuals with reproductive disorders, carriers of inversions accounted for 11% (3 out of 27) and 16% (5 out of 32), respectively, while in disease-defined patients − 21% (3 out of 14), and in prenatal diagnoses − 24% (8 out of 34). Among miscarriages, carriers of inversion were not found.

#### Involvement of single chromosomes in rearrangements

Data on the involvement of single chromosomes according to types of Rea, in disease-defined carriers and in asymptomatic carriers are summarized in Tables 3 and 4.

**Table 3:** Distribution of chromosomes according to type of rearrangement in affected carriers, including affected carriers of gonadal mosaicism ^a^, *n* = 252

Chromosome	Deletion *n* = 79	Duplication *n* = 47	Ring ^b^		Unbalanced translocation *n* = 30	Other unbalanced Rea *n* = 30	Unbalanced Rea total *n* = 238	Balanced Rea *n* = 14
			Unbalanced *n* = 30	Balanced *n* = 22				
1	1	6	0	0	1	1	9	3
2	2	2	0	3	2	0	9	2
3	3	3	0	0	3	4	13	1
4	4	1	3	2	1	1	12	3
5	2	2	0	0	4	2	10	0
6	1	0	0	2	4	0	7	3
7	6	2	0	1	2	3	14	1
8	6	2	0	2	5	4	19	1
9	0	0	2	0	6	0	8	1
10	0	0	0	0	4	1	5	0
11	6	3	0	0	1	0	10	1
12	3	7	0	1	2	4	17	2
13	8	1	4	1	3	0	17	0
14	5	3	0	1	1	3	13	1
15	4	4	1	1	4	2	16	1
16	1	1	0	1	2	0	5	0
17	4	6	0	2	0	0	12	2
18	13	1	9	2	7	5	37 (14%)	0
19	3	0	0	3	1	1	8	1
20	2	2	0	0	2	1	7	0
21	0	1	3	0	0	0	4	0
22	5	0	8	0	3	0	16	1
Total	79	47	30	22	58	32	268	24

^a^Excluding 13 cases of del 13q associated with rethinoblastoma and 22 cases of corrected rearrangements; ^b^excluding one unclear case

**Table 4:** Distributions of chromosomes according to type of rearrangement in asymptomatic carriers, *n* = 130

Chromosome	Deletion *n* = 30	Duplication *n* = 10	Ring *n* = 12^a^	Unbalanced translocation *n* = 7	Other unbalanced Rea *n* = 7	Unbalanced Rea, total *n* = 66	Balanced Rea *n* = 64
1	1	0	0	0	0	1	8
2	0	1	0	0	1	2	12
3	0	0	0	0	0	0	6
4	0	2	2	0	0	4	7
5	6	1	0	2	0	9	9
6	2	0	0	1	1	4	4
7	0	0	0	0	0	0	6
8	3	1	0	2	0	6	3
9	0	0	0	0	0	0	10
10	0	1	0	0	0	1	5
11	1	0	0	1	0	2	5
12	0	0	0	0	1	1	6
13	4	1	0	1	1	7	4
14	0	0	2	0	2	4	6
15	3	0	0	0	0	3	4
16	1	0	0	0	0	1	4
17	2	1	1	0	1	5	2
18	0	1	1	1	0	3(4%)	4
19	0	0	1	1	0	2	4
20	4	0	0	0	1	5	5
21	2	1	2	2	0	7	6
22	1	0	3	3	0	7	5
Total	30	10	14	12	8	74	125

^a^Breakpoints were not specified in 50% on the cases

The analysis showed that the compared groups differ statistically significantly in this indicator. In disease-defined patients, chromosome 18 is affected significantly more likely (14%) in comparison to other chromosomes. In addition, there is a difference in the involvement of chromosomes in various types of Reas. For example, chromosome 18, being the most frequent among both deleted chromosomes and ring chromosomes (13 and 11 cases, respectively), was found to have few duplications (1 case) and no involvement in balanced translocations. In contrast, chromosome 1 is more frequently found to be duplicated than deleted (6 cases vs. 1). Chromosome 21 appeared to be the least affected, with only 4 of 246 instances (1.5%).

In asymptomatic carriers, the distribution is statistically significantly different from that in carriers with clinical manifestations, at *p* = 0.003. Chromosomes most frequently involved in unbalanced Reas are chromosome 5 (11%), chromosome 13, chromosome 21, and chromosome 22 (10% each).

Rearrangements in chromosome 3, chromosome 7, and chromosome 9 are the most rarely detected (0% each). The rate of involvement of chromosome 18 (4.2%) does not differ from the expected figure of 4.5%. In prenatal diagnoses (Table 5), the distribution of involved chromosomes is more homogenous, with an apparent prevalence of chromosome 18 (13%).

**Table 5:** Distributions of chromosomes according to type of rearrangement in prenatal diagnoses, *n* = 130

Chromosome	Deletion *n* = 38	Duplication *n* = 13	Ring *n* = 16^a^	Unbalanced translocation *n* = 11	Other unbalanced Rea *n* = 18	Unbalanced Rea, total *n* = 96	Balanced Rea *n* = 34
1	2	4	0	3	0	9	6
2	2	0	0	0	0	2	9
3	0	1	0	1	0	2	3
4	4	1	0	1	0	6	4
5	2	0	0	2	0	4	4
6	1	0	0	0	1	2	5
7	1	1	0	1	1	4	3
8	1	0	0	0	0	1	1
9	0	0	0	2	0	2	3
10	3	1	0	0	0	4	3
11	4	1	0	0	2	7	1
12	2	2	1	1	1	7	4
13	2	0	1	1	3	7	2
14	0	0	0	3	1	4	1
15	1	0	2	3	2	8	1
16	2	0	0	0	1	3	4
17	0	2	0	1	0	3	2
18	8	0	4	0	2	14 (13%)	0
19	0	0	2	0	0	2	0
20	0	0	2	0	1	3	1
21	0	0	2	1	1	4	0
22	3	0	2	0	2	7	2
Total	38	13	16	20	18	105	59

^a^Breakpoints were not specified in 56% of the cases

Such analysis is of potential significance for the evaluation of the fitness of mosaic preimplantation embryos. It might be possible that rearrangements of certain chromosomes (for example, deletion of chromosome 18) are not tolerated by the embryo while others, being involved in segmental mosaicism (for example, chromosomes 5 and 21), might have good prospects. Again, we would like to stress that more cases should be collected for such a study.

When comparing the data of our study to the spectrum of chromosomal abnormalities with those obtained from preimplantation diagnostics (Coll et al. 2020), in both, it is noted that the most common chromosomal abnormality was deletion. However, we did not find exceptionally high involvement of chromosomes 1, 5, and 9, in contrast to the data presented by Coll et al. (2020).

Moreover, our data on the frequency of involvement in chromosome rearrangements as a function of their length contradicts the findings of Munné and Wells (2017) and Coll et al. (2020). They reported the predominant involvement of large chromosomes in mosaic unbalanced rearrangements in preimplantation embryos. According to our data (Table 6), large chromosomes are not more likely than short chromosomes to be involved in unbalanced Reas, in both disease-defined patients (_6_ 11 _20_ vs. _7_ 13 _25_) and in asymptomatic carriers (_1.3_ 2.4 _4.8_ vs. _4_ 9 _23_), as well as in prenatal diagnoses (_2.2_ 4.0 _7.7_ vs. _2.8_ 5.2 _11_). Conversely, large chromosomes tend to be involved in balanced Reas more often than short ones, both in disease-defined carriers (_0.7_ 1.5 _31_ vs. _0.2_ 0.6 _1.6_) and in asymptomatic carriers _(2.8_ 6.5 _12_ vs. _2.2_ 4.2 _8.6_), as well as in prenatal diagnoses (_2.0_ 3.7 _7.1_ vs. _0.6_ 1.3 _2.9_).

**Table 6:** Involvement of chromosomes in rearrangements according their size and type of rearrangement

Studies groups	Unbalanced rearrangements				Balanced rearrangement			
	Large chromosomes		Short chromosomes		Large chromosomes		Short chromosomes	
	Number of carriers (*N*_l_)	Rate (*N*_l_/12)	Number of carriers (*N*_s_)	Rate (*N*_s_/10)	Number of carriers (*N*_l_)	Rate (*N*_l_/12)	Number of carriers (*N*_s_)	Rate (*N*_s_/10)
Disease-defined carriers	133	_6_ 11 _20_	135	_7_ 13 _25_	18	_0.7_ 1.5 _3.1_	6	_0.2_ 0.6 _1.6_
Prenatal diagnoses	50	_2.2_ 4.0 _7.7_	55	_2.8_ 5.2 _11_	46	_2.0_ 3.7^a^_7.1_	13	_0.6_ 1.3^a^_2.9_
Asymptomatic carriers	30	_1.3_ 2.4 _4.8_	44	_4_ 9 _23_	81	_2.8_ 6.5 _12_	44	_2.2_ 4.2 _8.6_

^a^Difference between rates of involvement is statistically significant, mid-*p* = 0.039

Besides, some increase in the involvement of chromosomes 1, 9, and 5 in balanced Rea was observed in the group of asymptomatic carriers. However, it is reasonable to suggest that mosaicism for balanced Reas was not a matter of concern in preimplantation diagnostics; moreover, it is not readily identified by molecular technologies.

### Extent of mosaicism for unbalanced rearrangement

The proportion of cells with Reas, in addition to the number of cells studied, was not specified in all published cases; therefore, only individuals with a high (≥ 50%) frequency of cells with Reas were analyzed. In the disease-defined cohort, such individuals were found more often than among asymptomatic carriers: _43_ 47 _50_% (119 of 235) versus _16_ 23 _30_% (8 of 52); the difference is highly statistically significant since confidential intervals are not overlapped. In affected carriers of gonadal mosaicism, a high proportion of cells with an unbalanced Rea was detected even more frequently, in _35_ 59 _80_% (9 out of 15).

In prenatal diagnoses, the proportion of amniocytes with unbalanced Rea was reported in 66 cases. A high frequency of abnormal cells was found in 20 out of 39 (_42_ 53 _64_%) fetuses with an unfavorable pregnancy outcome and in 7 out of 27 (_18_ 27 _26_%) fetuses with a normal pregnancy outcome. Interestingly, the majority of affected carriers of balanced Rea (11 out of 13) showed low levels of mosaicism.

We believe that these data are in good accordance with reported results (Spinella et al. 2018; Viotti et al. 2020): mosaic embryos with an aneuploidy rate of < 50% had more favorable clinical outcomes than those containing > 50% aneuploidy. However, other authors reported that the degree of trophectoderm mosaicism was a poor prediction of ongoing pregnancy and miscarriage (Kushnir et al. 2018; Victor et al. 2019; Viotti et al. 2020). Moreover, Popovic et al. (2020) claimed to estimate the precise degree and prevalence of mosaicism based on a single biopsy to be conceptually unachievable.

### Sex ratio among carriers of segmental mosaicism

Data on SR in studied cohorts, according to two general categories, as carriers of unbalanced Rea and carriers of balanced Rea are presented in Table 7. It is evident that in almost all groups, there is a female predominance among carriers of unbalanced Rea, unlike the slight male prevalence among carriers of balanced Rea, both prenatally diagnosed and postnatally disease-defined, as well as among carriers with reproduction disorders. Unlike this general trend, miscarriages and transmitting carriers of balanced rearrangement demonstrate apparent female predominance.

**Table 7:** Sex ratio in various cohorts of segmental mosaicism carriers

Study groups		Unbalanced rearrangements				Balanced rearrangements			
		Males	Females	Sex ratio	*p*	Males	Females	Sex ratio	*p*
					SR = 1.06				SR = 1.06
Affected carriers	Disease-defined carriers of somatic mosaicism	102	121	_0.6_ 0.8 _1.1_	0.094	8	6	_0.5_ 1.3 _3.7_	0.79
	Affected carriers of somatic/gonadal mosaicism	2	14	_0.0_ 0.2 _0.6_	0.0018	0	0	—	—
	Mid-*p*	0.006				0.5			
Prenatal diagnoses		45	51	_0.6_ 0.9 _1.3_	0.41	19	15	_0.6_ 1.3 _2.5_	0.76
Fetal deaths		7	17	_0.2_ 0.4 _1.0_	0.039	1	7	_0.0_ 0.3 _0.9_	0.034
Mid-*p*		0.14				0.033			
Asymptomatic carriers	Transmitting carriers and carriers identified by chance	11	41	_0.1_ 0.3 _0.5_	10^−5^	12	20	_0.3_ 0.6 _1.2_	0.16
	Carriers with recurrent miscarriage	2	6	_0.1_ 0.4 _1.5_	0.17	12	10	_0.5_ 1.0 _2.2_	0.83
	Carriers with infertility	3	3	_0.2_ 1.0 _4.4_	1	8	2	_0.9_ 3.2 _16_	0.11
	Mid-*p*	0.28				0.053			

Data from the more detailed analysis of SR among carriers with clinical abnormalities according to the specific types of Rea are presented in Table 8. A notable predominance of female individuals was already stated in Table 7. This shift appears to be mainly due to the contribution of Reas that cause the loss of chromosomal material; in this collective group including deletions, apparently deleted rings, etc., 47♀/85♀ were found, the SR is _0.39_ 0.55 _0.79_. Also, a deficit of males is observed among carriers of Reas with gain/loss of chromosomal material: 15♂/21♀ (SR = _0.37_ 0.72 _1.4_). But for Reas characterized by a gain of chromosomal material, some predominance of males is noticeable: 46♂/36♀, SR = _0.8_ 1.3 _2.0_. Among the carriers of balanced Reas (including ring chromosomes without deletions), there are slightly more males: 21♂/17♀, SR = _0.7_ 1.2 _2.3_. Among fetuses with unbalanced Rea, there are slightly more female carriers: 45♂/ 51♀, SR = _0.6_ 0.9 _1.3_. Overall, carriers of unbalanced Rea (referred for genetic testing as a result of special indications including the abnormal US) are characterized by poor pregnancy outcomes (Table 8). A favorable pregnancy outcome was more often observed in male carriers: 21♂/11♀, SR = _0.9_ 1.9 _3.9_. Among unfavorable outcomes, female carriers predominated: 20 males/29 females, SR = _0.4_ 0.7 _1.2_, the difference is statistically significant at mid-p = 0.032. Proportions of abnormal outcome are practically evenly distributed over various Rea types (Table 9), which might be explained by small sample sizes.

**Table 8:** Sex of affected carriers (postnatal disease-defined and affected carriers of gonadal mosaicism) according to types of rearrangements

Type of rearrangement		Carriers’s sex		Total
		Males	Females	
Deletions	Excluding del(13) associated with retinoblastoma	27	52	79
	del(13) associated with retinoblastoma	5	8	13
Duplications		25	22	47
Rings	Apparently deleted	11	19	53
	No apparent deletion	12	10	
	Uncertain	1		
Unbalanced translocations	Loss	1	1	30
	Gain	7	6	
	Gain/loss	7	8	
Other unbalanced rearrangements	Loss	1	2	30
	Gain	7	3	
	Gain/loss	5	12	
Apparently balanced rearrangements	Inversions	2	1	14
	Reciprocal translocations	6	5	
Rescued rearrangements ^a^	Loss	2	3	22
	Gain	7	5	
	Gain/loss	3	1	
	Balanced	1		
Total		130	158	288

^a^Including 3 deletions, 3 duplications, 2 rings, 12 unbalanced translocations, and 2 other unbalanced rearrangements

**Table 9:** Pregnancy outcomes according to type of unbalanced rearrangement and fetuses’ gender

Type of rearrangement	Pregnancy outcomes					
	Abnormal		Normal		Total	Proportion of abnormal outcome, %
	Males	Females	Males	Females		
Deletion	11	10	9	5	35	_44_ 60 _75_
Duplication	2	5	2	1	10	_43_ 70 _90_
Ring	2	4	4	1	11	_31_ 55 _83_
Unbalanced translocation	1	4	1	2	8	_30_ 62 _86_
Other rearrangement	4	6	5	2	17	_51_ 59 _79_
Total	20	29	21	11	81	_51_ 60 _72_

It is interesting to note that in prenatal diagnoses, as well as in other studied cohorts (except miscarriages), male patients predominated among the carriers of inversions. Among 7 prenatally diagnosed carriers of inversion, 6 were males. Overall, the SR among inversion carriers was _1.0_ 2.8 _7.4_ (14♂/5♀). Carriers of balanced Rea diagnosed prenatally are usually born normal. Of the 29 fetuses with reported outcomes, only four (2♂/2♀) were born with anomalies. In this group, as well as among postnatally diagnosed carriers, with or without clinical manifestations, there is a typical predominance of males: 19♂/15♀.

Analysis of data from cytogenetic surveys of fetal deaths revealed a female prevalence among carriers of unbalanced SM: 8♂/18♀ in cases where the likelihood of contamination with maternal cells was either excluded or being very small, and 8♂/24♀ in the total sample.

While sex ratio in the general population is considered paramount genetic, medical, and social essence, the association of SR deviations with chromosome abnormalities is still apparently underappreciated. Previous studies demonstrated SR may be considered an effective tool for recognition and examination of pathologic processes, and risks prediction. For example, the predominance of the females, found among carriers of rearrangements with breakpoints in the pericentromeric regions, was explained by sex-specific instability of pericentromeric regions as the earliest manifestation of sexual dimorphism (Kovaleva and Shaffer 2003). SR-based studies suggested low-level mosaicism for a normal cell line in homologous Robertsonian translocation carriers which would alter their reproductive options (Kovaleva 2007), indicated that gender affects clinical suspicion of Down syndrome (Kovaleva 2011), and suggested an impact of paternal rearrangement on maternal chromosomes’ segregation after fertilization (Kovaleva 2013)]. Recently, the predominance of female individuals among newborn carriers of non homologous Robertsonian translocations was discovered, which was explained by the mechanism of sex-specific correction of the initial trisomy. Uniparental disomy resulting from trisomy correction was female-biased too (Kovaleva 2017). Therefore, we opted to apply the examination of SR as a useful tool when studying the epidemiology of segmental mosaicism, though the unusual sex ratios among carriers of SM due to noncentromeric breakpoints were not readily predictable.

What can explain the excess of female individuals among carriers of unbalanced SM, which is especially pronounced in asymptomatic carriers? At least three mechanisms can be considered: sex-specific (inherent in female embryos) genomic instability, intrauterine selection of male carriers, or sex-specific (inherent in male embryos) elimination of abnormal cells.

Poszygotic instability of the female genome as an explanation for the shift in the sex ratio presupposes the prevalence of female individuals also among carriers of balanced rearrangements. However, in almost all studied groups, among the carriers of balanced rearrangements, males predominated, and the sex ratio is close to the population value. The high intrauterine mortality of male fetuses can also be excluded from consideration since, among fetal deaths with segmental mosaicism, a clear predominance of female abortions was observed.

The results of our previous and present studies suggest sex-specific elimination of chromosomal abnormalities. It was reported that early female embryos develop somewhat more slowly than male embryos (Pergament et al. 1994; Alfarawati et al. 2011), possibly due to the process of X chromosome inactivation, which occurs at the stage of ≤ 10 cells (Pergament et al. 1994). More active proliferation of male cells can facilitate the efficient elimination of the abnormal cell line. It can be assumed that the age of the mother influences the rate of elimination of abnormal cells. The discovered phenomenon deserves further study, including a comparative analysis with the age of the parents of carriers of asymptomatic mosaicism. Unfortunately, such data are practically absent in the literature. Studies of the parental origin of rearranged chromosomes would be of considerable theoretical interest.

Sex ratio in asymptomatic carriers suggested that the prognosis for male carriers of segmental mosaicism for normal development and reproductive health is more favorable than for female carriers. This assumption is supported by data on the sex ratio among fetuses with a normal pregnancy outcome. However, it should be borne in mind that the male cells, apparently, are tolerant to an excess of chromosomal material, and such an anomaly as duplication may “go unnoticed.”

### Parental ages of segmental mosaicism carriers

Maternal age was known for 147 disease-defined patients and paternal age for 120 patients. It is noteworthy that the maternal age of female carriers of almost all types of Reas appeared to be higher than in mothers of male carriers. Overall, the mean maternal age of 83 female carriers was _27_ 29 _29_ years and of 64 male carriers _24_ 25 _27_ years. The proportion of women of 35 years and older is _12_ 21 _32_% and _3_ 7 _15_%, correspondingly. The difference between distributions is statistically significant at *p* < 0.0092.

At the same time, no difference in the paternal age depending on the sex of the abnormal offspring was found: in both male and female groups, the paternal average age was similar, 29.7 and 30 years, correspondingly. The sex ratio displays an apparent tendency to decrease with the increase of maternal age from 3.2 in the group < 20 years to 0.2 in the group aged 40 years and older (Table 10), with an exact *p*-value for the trend of 2^.^10^−4^. No such trend was found when analyzing SR according to paternal ages.

**Table 10:** Sex ratio in disease-defined carriers according to maternal age

Age groups, years	Male carriers		Female carriers		Sex ratio (*P*_M_/*P*_F_)
	Number	Proportion (*P*_M_), %	Number	Proportion (*P*_F_), %	
< 20	8	_5_ 13 _26_	2	_0_ 2 _10_	_0.9_ 3.2 _15.6_
20–24	20	_18_ 31 _28_	18	_11_ 22 _35_	_0.6_ 1.1 _2.1_
25–29	25	_24_ 39 _56_	27	_20_ 33 _47_	_0.5_ 0.9 _1.6_
30–34	7	_4_ 11 _25_	19	_12_ 23 _37_	_0.16_ 0.39 _0.86_
35–39	3	_1_ 5 _16_	11	_6_ 13 _25_	_0.08_ 0.32 _0.93_
≥ 40	1	_0_ 2 _11_	6	_2_ 7 _18_	_0.03_ 0.25 _1.1_
Total	64	100	83	100	_0.6_ 0.8 _1.1_
Exact *p*-value for homogeneity			0.0092		
Exact *p*-value for trend					2·10^–4^

There is no consensus in the literature regarding parental age as a factor influencing embryo segmental mosaicism. Some indicate that advanced maternal age was not seen to be correlated with a higher prevalence of mosaicism (Munné and Wells 2017; Nakhuda et al. 2018). Other reports observed a significant maternal age effect on the success of mosaic embryos (Victor et al. 2019; Greco et al. 2020), while Coll et al. (2020), found that a positive association with mosaicism showed only paternal age. Our data demonstrate meaningful differences in the maternal age distributions between disease-defined males and females, while paternal ages did not differ. This data cannot be compared with that obtained from studies on pre-implantation embryos, since these reports persist in ignoring such important genetic parameters as gender. This, unfortunately, is typical for research on prenatal diagnostics and fetal death. We would propose the desirability of consensus protocols for the presentation of survey data submitted to publication, including information about gender, parental ages, and reproductive history.

## Conclusions

Our study showed that the type of rearrangement, the chromosomes involved, the frequency of abnormal cells, the gender of the carrier, and the maternal age may be factors influencing the clinical manifestation of chromosomal imbalance. The introduction of molecular methods for the diagnosis of chromosomal abnormalities will inevitably cause an increase in the number of diagnosed cases of segmental mosaicism and, accordingly, the number of challenges in genetic counseling. Consequently, the general goal of future studies should be not just an increase in the amount of knowledge useful for medical and genetic counseling of families of carriers of segmental mosaicism, but the development of algorithms for optimization of the prognosis. This requires representative samples of carriers of segmental mosaicism, together with the registration of all known significant/potentially significant variables. Therefore, we emphasize the need to publish every single detected case of segment mosaicism and invite colleagues to join the international consortium “Segmental mosaicism: determination of factors affecting the clinical manifestation of chromosomal imbalance” (see Research Gate).

## Supplementary Information

Below is the link to the electronic supplementary material.

Supplementary file1 (RAR 3855 KB)

## Data Availability

Data sharing not applicable to this article as no datasets were generated or analyzed during the current study.
